# Protective Effect of Natural Antioxidants on Reducing Cisplatin-Induced Nephrotoxicity

**DOI:** 10.1155/2022/1612348

**Published:** 2022-11-14

**Authors:** Jie Zhou, Run-cong Nie, Yi-xin Yin, Xiao-xia Cai, Dan Xie, Mu-yan Cai

**Affiliations:** ^1^State Key Laboratory of Oncology in South China, Collaborative Innovation Center for Cancer Medicine, Sun Yat-sen University Cancer Center, Guangzhou, China; ^2^Department of Gastric Surgery, Sun Yat-sen University Cancer Center, State Key Laboratory of Oncology in South China, Collaborative Innovation Center for Cancer Medicine, Guangzhou, China; ^3^Department of Pathology, Sun Yat-sen University Cancer Center, State Key Laboratory of Oncology in South China, Collaborative Innovation Center for Cancer Medicine, Guangzhou, China

## Abstract

The clinical application of cisplatin is limited by its adverse events, of which nephrotoxicity is the most commonly observed. In a cisplatin-induced pathological response, oxidative stress is one of the upstream reactions which inflicts different degrees of damages to the intracellular material components. Reactive oxygen species (ROS) are also one of the early signaling molecules that subsequently undergo a series of pathological reactions, such as apoptosis and necrosis. This review summarizes the mechanism of intracellular ROS generation induced by cisplatin, mainly from the consumption of endogenous antioxidants, destruction of antioxidant enzymes, induction of mitochondrial crosstalk between the endoplasmic reticulum by ROS and Ca^2+^, and destruction of the cytochrome P450 (CYP) system in the endoplasmic reticulum, all of which result in excessive accumulation of intracellular ROS and oxidative stress. In addition, studies demonstrated that natural antioxidants can protect against the cisplatin-induced nephrotoxicity, by reducing or even eliminating excess free radicals and also affecting other nonredox pathways. Therefore, this review on the one hand provides theoretical support for the research and clinical application of natural antioxidants and on the other hand provides a new entry point for the detailed mechanism of cisplatin nephrotoxicity, which may lay a solid foundation for the future clinical use of cisplatin.

## 1. Introduction

Cisplatin (*cis*-diamminedichloroplatinum (II), CDDP) is one of the most widely used antitumor drugs around the world, especially in the treatment of many solid tumors, such as uterine cancer [1], testicular cancer [2, 3], ovarian cancer [4, 5], bladder cancer [6], head and neck cancer [7], and lung cancer [8]. A study even showed that cisplatin is effective in treating testicular cancer, with a curative rate reaching up to 90% or higher [3]. Cisplatin induces the cross-linking of intra- and interchain DNA which can destroy the replication and transcription of DNA and induce DNA damage [9, 10]. Mildly damaged DNA can be repaired whereas extensive DNA damage can lead to the irreversible injury and cell death, which is the fundamental mechanism of cisplatin's cytotoxic antitumor activity in cancer therapy; proliferating cancer cells are sensitive to DNA damage especially [11, 12]. Since cisplatin has been used in antitumor treatment, it has attracted much attention not only because of its curative effect but also due to its potential side effects such as gastrointestinal reactions, renal toxicity, neurotoxicity, and ototoxicity [13, 14]. Studies have demonstrated that the main dose-limiting side effect is renal toxicity [15–18].

The renal toxicity induced by cisplatin greatly limits its dosage and time to treatment. It is recognized that 25–35% of patients experience a significant reduction in renal function after the administration of a single dose of cisplatin [19]. Direct effects on the renal vasculature are involved; however, cisplatin nephrotoxicity mostly shows a tubular damage pattern of dysfunction and disorder, producing electrolytic disturbances, acute tubular injury (ATI), and acute kidney injury (AKI), with elevated plasma creatinine and urea levels [20, 21], which may occasionally cause a chronic fibrotic nephropathy finally. Although protective approaches have been shown, such as hydrotherapy in the clinical practice, the incidence of cisplatin-induced nephrotoxicity is still high [22]. Therefore, understanding the mechanism of cisplatin-induced nephrotoxicity may help investigate novel renoprotective interventions and provide better protection against cisplatin-induced nephrotoxicity.

Treatment safety is always a primary consideration in studies involving human subjects, and the ideal protective component should be nontoxic, easily available, effective at lower doses, and economical. In recent years, because of the potential ability to suppress cancers and reduce the incidence of cancer, natural dietary agents have drawn a great deal of attention from researchers and the general public [23]. In addition, the combinational use of natural antioxidant and chemotherapeutic drugs may help mitigate drug-associated toxicities [24]. Also, the antioxidant activity of natural antioxidants indicated that it could serve as a potential agent to treat oxidative stress-related cellular pathology, such as cisplatin-induced nephrotoxicity. Therefore, in this review, we summarized the up-to-date studies to comprehensively explore whether antioxidants can be used for renoprotection against cisplatin injury.

## 2. Accumulation of ROS Is the Upstream Reaction Leading to Pathological Events in Cisplatin-Induced Nephrotoxicity

For over a decade, oxidative stress has been regarded as an important factor that contributes to cisplatin-induced nephrotoxicity [25, 26]. Researches showed that cisplatin mainly causes renal damage by inducing oxidative stress in renal tubular and glomerular cells, thereby leading to cell necrosis and apoptosis, vascular dysfunction, and strong immune response [27–31]. In recent years, the mechanism of apoptosis inducing renal tubular cell dysfunction has been a focus of investigation of cisplatin nephrotoxicity. Several pathways of apoptosis have been implicated; the first one is the extrinsic pathway mediated by death receptors, such as Fas and tumor necrosis factor-*α* (TNF-*α*) receptor [32]; the intrinsic pathway centered on mitochondria, which relates to cellular stress that leads to the activation of the proapoptotic Bcl-2 family proteins Bax and Bak on the mitochondrial membrane [33, 34], and the endoplasmic reticulum (ER) stress pathway, which is activated by caspase-12 mainly [35]. Although the progression of apoptosis induced by cisplatin results from several precise mechanisms, oxidative stress has been widely related to them; furthermore, the relationship between oxidative stress and apoptosis might be direct as they share a similar dysregulation of mitochondria.

Oxidative stress is a cellular stress response when the reduction and oxidation (redox) balance between reactive species and antioxidant species is disrupted. Oxidation is defined as the chemical reaction, which is an important part of cellular metabolism and other biological function that allows electrons to be separated from an atom or molecule. Free radicals are atoms or molecules that have unpaired electrons and are always unstable and highly reactive, of which excess accumulation usually induces oxidative stress. There are two common types of free radicals: oxygen-derived radicals, also called reactive oxygen species (ROS), and nitrogen-based radicals, also called reactive nitrogen species (RNS). ROS can be grouped into oxygen-centered radicals, including the superoxide anion (O2^•^-), hydroxyl radical (•OH), alkoxyl radical (RO•), and peroxyl radical (ROO•), and oxygen-centered nonradicals, which are also named as nonfree radical oxygen intermediates, such as hydrogen peroxide (H_2_O_2_) and singlet oxygen (^1^O_2_) [36, 37]. All cells in the body are exposed chronically to free radicals. Antioxidants are present in serum and erythrocytes, as well as other tissues and organs to prevent damage caused by ROS, RNS, and other free radicals. The system of antioxidant is composed of molecules having antioxidant properties such as glutathione (GSH), vitamin C, transferrin, albumin, and various antioxidant enzymes, such as glutathione peroxidase (GSH-Px) [38]. Antioxidant defense in the human body is weakened; therefore, oxidative stress occurs when the delicate balance between the amounts of oxidants and antioxidants is broken.

It is worth noting that once cisplatin enters kidney cells, it rapidly induces a large accumulation of ROS in the cell, breaks the balance of the redox system in the intracellular environment, and contributes to the out leakage of several intracellular compositions that could cause different degrees of damage. ROS can also target and modify multiple molecules in the cells, such as lipids, proteins, and DNA, which can result in cellular stress [28, 39]. In addition, ROS appear to be involved in the activation of several important signaling pathways during cisplatin-induced nephrotoxicity. Some studies have confirmed that the massive accumulation of ROS and other mitochondrial oxidative stress-induced functional disorders is the early signaling of cisplatin-induced pathological events, such as inflammatory response and several apoptotic signal pathways mediated by caspases and mitogen-activated protein kinases (MAPKs) [40–45]. Meanwhile, researches show that other stress like nitrosative stress produces a toxic role in cisplatin-induced nephrotoxicity by another free radical, RNS [46, 47], and also indicate that nitrosative stress and oxidative stress form a quantity of steps in the mechanism of cell damage together [39, 48, 49]. However, this review mainly focuses on oxidative stress and ROS and discusses the mechanism of cisplatin-induced oxidative stress response that contributes to nephrotoxicity.

## 3. How Does Cisplatin Induce Accumulation of ROS and Cause Oxidative Stress Response?

**Figure 1:**
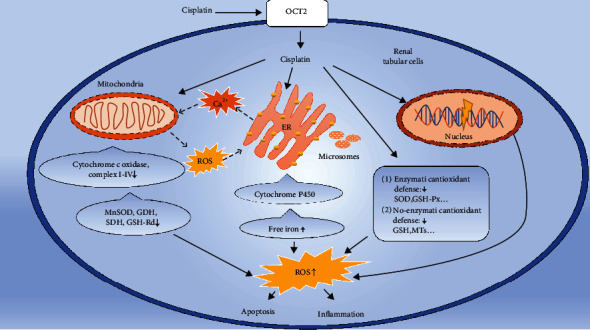
The major mechanism of excessive ROS generation induced by cisplatin in renal tubular cell. →: enhancement; ⇢: circle line. The mechanism of oxidative stress induced by cisplatin is mainly from the consumption of endogenous antioxidants, destruction of antioxidant enzymes, and induction of mitochondrial damage and makes the crosstalk between mitochondria and endoplasmic reticulum (ER) by ROS and Ca^2+^, destructs the CYP 450 system, then releases the free iron from ER, microsomes, etc., then makes the excessive accumulation of ROS in renal tubular cell, and finally results in apoptosis and inflammation. ROS: reactive oxygen species; GSH: glutathione; GSH-Px: glutathione peroxidase; SOD: synthesize superoxide dismutase; MTs: metallothionein; CYP 450: cytochrome P450; MnSOD: manganese-containing superoxide dismutase; GDH: glutamic dehydrogenase; SDH: succinate dehydrogenase; GSH-Rd: GSH-reductase.

As mentioned above, excess ROS are harmful due to their oxidant species and might cause cellular dysregulation and damage to several cell structures. In this review, we classified the oxidative stress as follows: (a) lipid peroxidation assessed by the determination of malondialdehyde (MDA), 4-hydroxyno-nenal (4-HNE), thiobarbituric acid reactive substances, or other lipid peroxide content; (b) 8-hydroxy-deoxy-guanosine (8-OHdG), an oxidatively modified DNA adduct; and (c) GSH and other common antioxidant depletion [39]. The major mechanisms of excessive ROS accumulation induced by cisplatin are mainly caused by the following detailed approaches (Figure 1):

1. Since the uptake by renal tubular epithelial and glomerulus cell, cisplatin can rapidly reduce the endogenous antioxidant substances, such as GSH and metallothionein (MTs). Although the exact mechanism of cisplatin reducing endogenous antioxidant is still unclear, cisplatin may play a role in the following ways: (a) cisplatin can reduce copper and zinc in the cell. It is acknowledged that copper and zinc are the necessary substances to synthesize superoxide dismutase (SOD) in the cytoplasm and mitochondrial inner membrane and to help take in selenium, which is necessary for the synthesis of GSH-Px [50–52]. It is known that SOD and GSH-Px are the most important enzymes that can regulate and maintain the balance of redox reactions in the cell. Therefore, cisplatin destroys the first line of defense, that is, enzymatic antioxidant defense response. When the corresponding SOD and GSH-Px are reduced, the free radical content in the body rises sharply and the redox balance is destroyed; then, oxidative stress is formed. (b) Cisplatin can directly combine with thiol-containing compounds such as GSH, MTs, GSH-Px, and SOD, resulting in a direct reduction of endogenous reducing substances that can scavenge the ROS [52–54], such as ascorbic acid and GSH; thus, cisplatin can destroy the second line of defense, that is, nonenzymatic antioxidant defense response [51]. When endogenous antioxidants are consumed excessively, the cell's redox equilibrium state is broken, which triggers the oxidative stress response and causes damage to cells and even tissues
2. After being transported into the cell, cisplatin enters into mitochondria and binds to mitochondrial DNA, causing mitochondrial dysfunction and increasing ROS production via the disrupted respiratory chain, which may be more important than nuclear DNA damage in cisplatin-induced cell death [55]. Cisplatin affects the normal transmission of electrons on the oxidative respiratory chain and produces a large number of free radicals that can be generated to form ROS. Meanwhile, the decrease in cytochrome c oxidase (COX) activity and the low expression of complex I-IV protein can result in ROS generation [56]. It is also reported that complex III and glycerol 3-phosphate dehydrogenase can release superoxide into the intermembrane space, suggesting that the release of mitochondrial ROS into the cytosol is important to cellular damage [57], especially the damage to endoplasmic reticulum (ER) which will result in a vicious circle between mitochondria and ER. In normal condition, calcium (Ca^2+^) in mitochondria can reduce ROS when complexes I and III are working as usual but can increase the ROS generation when these complexes were inhibited by pharmacological agents [58]. Since ER is a major site of calcium storage which can cause an increased release of Ca^2+^ to mitochondria, when mitochondrial ROS releases into the cytosol and targets ER-based calcium channels, a vicious circle will finally develop [59]

Besides the effect of Ca^2+^ and ROS crosstalk between ER and mitochondria, cisplatin can also reduce the activity of manganese-containing superoxide dismutase (MnSOD), glutamic dehydrogenase (GDH), succinate dehydrogenase (SDH), and mitochondrial GSH-reductase (GSH-Rd) in mitochondria directly, resulting in a sharp reduction in mitochondrial-related endogenous antioxidants, which can cause dysregulation of mitochondrial antioxidant defense systems, and finally increase ROS accumulation and oxidative stress [3, 40, 60].

(3) Cisplatin increases the free iron by acting on the cytochrome P450 (CYP) system in the endoplasmic reticulum and microsomes, and iron ions are important catalysts in the production of ROS [61]. In the presence of iron ions and H_2_O_2_, the first and second steps of the Haber–Weiss reaction will cause the excessive generation of ·OH rapidly [62], resulting in more ROS accumulation and triggering oxidative stress response [63, 64]

## 4. The Protective Mechanism of Natural Antioxidants

**Figure 2:**
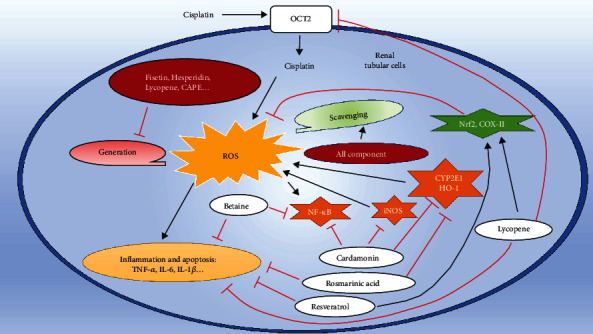
The major protective mechanisms of the representative natural antioxidants in cisplatin-inducing damage in renal tubular cell. →: enhancement; red left tack: inhibition. All the natural antioxidants have the ability to scavenge the accumulation of ROS induced by cisplatin. Some special can reduce the ROS generation, include Fisetin, Hesperidin, Lycopene, CAPE. Betaine, resveratrol, and cardamonin can also inhibit the inflammation of renal tubular cells by inhibiting the nuclear factor kappa-B (NF-*κ*B) or other inflammatory factors. Cardamonin and rosmarinic acid can inhibit oxidative stress by decreasing some molecules that can induce generation of ROS via nitric oxide synthase (iNOS), cytochrome P450 2E1 (CYP2E1), and heme oxygenase-1 (HO-1). Lycopene can reduce the generation of ROS, alleviate inflammation, and also reduce the uptake of cisplatin by downregulating the expression of organic cation transporters 2 (OCT2), which are only expressed in the proximal tubules. TNF-*α*: tumor necrosis factor-*α*; IL-6: interleukin-6; IL-1*β*: interleukin-1*β*.

Researches have shown that many natural products which may decrease various side effects induced by anticancer drug are available over the counter [24]. It is known that antioxidant is the substance that can put off, prevent, or twist the oxidative damage in a target molecule [65]. In addition, natural antioxidants are a group of compounds that naturally have the antioxidant properties, without excluding other biological properties [66]. Natural antioxidants can protect against mitochondrial dysfunction and inflammation [67–69]. Moreover, a large number of experimental studies showed that natural antioxidants can protect against cisplatin-induced nephrotoxicity [70]. Here, we summarized the protective mechanisms of natural antioxidants in cisplatin-induced nephrotoxicity. Besides, we used a variety of representative antioxidants to present the underlying mechanism (Figure 2 and Table 1).

### 4.1. Reducing the Production of ROS

Various experimental results showed that natural antioxidants can achieve protection by reducing the production of ROS. Among them, fisetin not only can reduce the migration and invasiveness of cervical cancer [71], but it can also significantly reduce the cisplatin-induced kidney damage by reducing the mitochondrial damage and ROS generation to alleviate tissue oxidative stress response [40]. Hesperidin [53], as well as lycopene [72] and caffeic ethanol extract (caffeic acid phenethyl ester, CAPE) [73], can reduce the production of ROS by restoring antioxidant enzyme activity (Table 1). The restored antioxidant enzymes can resist the production of ROS from the first line of defense, thereby protecting the kidney from damage caused by cisplatin.

### 4.2. Removing Excess ROS

After a review of relevant experimental results, a total of 12 natural antioxidants were identified to have the ability to remove excess ROS induced by cisplatin (see Table 1). Since hesperidin can chelate with divalent iron [74] and reduce the generation of ROS, it has a stronger ability to remove OH and peroxide than some synthetic antioxidants, such as vitamin C, butylated hydroxytoluene (BHT), and butylated hydroxyanisole (BHA). Moreover, other natural antioxidants, such as Origanum majorana [27], cardamonin [75], quercetin [76], naringenin [50], and resveratrol [77, 78], also have an effect on removing excess ROS. Betaine even can clear ROS by increasing the synthesis of endogenous antioxidant GSH in the body, thereby offering protection against cisplatin nephrotoxicity [52].

### 4.3. Other Nonredox Pathways That Protect the Kidney from Damage

It is worth noting that most natural antioxidants not only have their corresponding protective effects on reducing or removing ROS but also have certain ability to resist other damage mechanisms caused by cisplatin. For example, it is reported that betaine not only can reduce inflammatory effects by inhibiting the expression of interleukin-6 (IL-6) and tumor necrosis factor-*α* (TNF-*α*) [79, 80] but also can directly reduce kidney damage by inhibiting the activation of nuclear factor kappa-B (NF-*κ*B) and exogenous apoptotic pathways [52]. The combination of fisetin and cisplatin can also inhibit the activation of NF-*κ*B and inflammatory response to protect the kidney from damage [40]. Preclinical and clinical studies showed that hesperidin has the effects of antihypertensive, antihyperlipidemia, antioxidation, and anti-inflammatory [81]. Moreover, combined with cisplatin, hesperidin can reduce the nitrification stress response, lower the TNF-*α* content in plasma, and improve neutrophil infiltration in kidney tissue, thereby resulting in amelioration of cisplatin-induced kidney damage [53]. Cardamom also showed a strong anti-inflammatory effect, inhibiting the production of many inflammatory mediators, such as IL-1*β*, TNF-*α*, NF-kB, and inducible nitric oxide synthase (iNOS) [75]. Francescato et al. reported that quercetin can also inhibit the production of NF-*κ*B to protect against cisplatin-induced kidney damage [76]. Lycopene can also inhibit the production of NF-*κ*B and increase the expression of nuclear factor erythroid 2-related factor (Nrf2)/heme oxygenase-1 (HO-1), which is related to resist oxidative stress and inflammatory response [72]. Besides, lycopene can change the quantity and quality of transporters in the renal tubules to inhibit the uptake of cisplatin and improve the filtration rate of cisplatin in the kidney [82], and resveratrol can modulate cisplatin pharmacokinetics and lower its renal accumulation and increase its plasma half-life to protect the kidney [83].

In summary, natural antioxidants can reduce excess ROS because of their antioxidant properties. They can also achieve protective effects by restoring endogenous antioxidant enzyme functions and participate in the synthesis of endogenous antioxidants. Furthermore, most natural antioxidants show a certain resistance in the damage mechanism of nonredox reactions. These mechanisms have a certain significance for future research and clinical use.

## 5. Research Status of the Representative Natural Antioxidants Protecting against Cisplatin Nephrotoxicity

### 5.1. Hesperidin

Hesperetin is one of the most abundant natural flavonoids, which is rich in a large number of fruits and vegetables [84], and its glycoside form, hesperidin (hesperetin 7-rhammnoglucoside), is also abundant in the fruit peel of *Citrus aurantium* L. [85]. Studies showed that ingested as glycoside form, hesperidin is hydrolyzed by glycosidase to the bioactive component hesperetin and is then absorbed [86, 87]. Several *in vitro* and *in vivo* studies have shown that hesperetin displays numerous biological activities, such as antioxidant [88] and anti-inflammatory [89]. In addition, a phenotypic experiment reported that hesperidin has a neuroprotective effect in mice [90]. After that, more and more studies showed that hesperidin can also protect against cisplatin-induced nephrotoxicity. In 2014, Sahu et al. [53] found that hesperidin can suppress the generation of ROS and proinflammatory cytokine TNF-*α* and apoptosis/necrosis and inhibit lipid peroxidation and oxidative stress, which consequently protect against cisplatin-induced acute renal injury. In 2016, Kaltalioglu and Coskun-Cevher found that hesperidin can reduce renal toxicity caused by cisplatin and demonstrated that the reduction of ROS is related to the recovery of catalase (CAT) activity and the increase of GSH content. Moreover, the inflammatory effect caused by cisplatin can also be alleviated by hesperidin [91]. Subsequently, Chen et al. [92] used HK2 cells to explore the protecting mechanism of hesperetin and found that hesperetin may protect against nephrotoxicity caused by cisplatin by inhibiting NADPH oxidase 4 (Nox4) in a time- and dose-dependent manner, activating the Nrf2 antioxidant signaling pathway and activating Sirtuin 6 (SIRT6).

### 5.2. Cardamonin

Cardamonin (CDN), a chalcone found mainly in the seeds of Alpinia katsumadai (Caodoukou in Chinese), is a medicinal herb that has been widely used to treat several diseases for thousands of years [93]. It can increase cell apoptosis or autophagy in nasopharyngeal carcinoma, prostate cancer, triple-negative breast cancer cells, and colorectal carcinoma cells [94–97]. In addition, cardamonin may strengthen the anticancer activity of cisplatin in several cancer types, such as ovarian cancer, hepatocellular carcinoma, prostate cancer, and colon tumor cells [75, 98]. El-Naga found that the underlying mechanisms of cardamonin in the nephroprotective effect may be partially attributed to the significant increase in SOD and GSH, which can reduce the lipid peroxidation and cisplatin-induced oxidative stress [75]. In brief, these studies have shown that cardamonin can not only increase the anticancer effect of cisplatin but also reduce the renal toxicity of cisplatin. Therefore, cardamonin can have great clinical value.

### 5.3. Quercetin

Quercetin (QE) is a major class of polyphenolic flavonoid compounds. Quercetin is one of the most abundant flavonoids in the human diet and has many beneficial effects on human health, such as cardioprotection, anti-inflammatory, antiproliferative, and anticancer activities [99, 100]. As early as in 1998, Kuhlmann et al. reported that quercetin has an antitumor ability and can protect against kidney damage caused by cisplatin [101]. In 2011, Sanchez-Gonzalez et al. found that the nephroprotective effect of quercetin may be related to its antioxidant activity and its ability to inhibit renal inflammation and tubular cell apoptosis, which can protect against cisplatin-induced nephrotoxicity in rats. Moreover, quercetin protects the kidneys from renal toxic damage inflicted by cisplatin, without altering its therapeutic anticancer activity [102]. In 2015, Almaghrabi found that quercetin can obviously induce CAT, SOD, and GPx gene expressions, enhance their enzyme activities to reduce the oxidative stress caused by cisplatin, and reduce the ROS generation by decreasing the MDA and protein carbonyl levels [103]. In 2016, Li et al. also provided the evidence that quercetin can reduce nephrotoxic damage and potentiates the anticancer activity of cisplatin by decreasing crypt multiplicity, mainly because quercetin can reduce the number of aberrant crypt foci (ACF), which is a colon carcinoma precursor in human and rats [104]. Follow-up studies elaborated the same view that quercetin could reduce kidney damage caused by cisplatin by reducing ROS production, anti-inflammatory, and other effects [105, 106]. Similar to cardamonin, quercetin can reduce the renal toxicity of cisplatin, without decreasing the anticancer effect of cisplatin.

### 5.4. Naringenin

Naringenin (NG) is a dominating flavanone in citrus fruits and possesses a broad range of biological and pharmacological activities [107, 108]. Several studies have shown that naringenin has many protective effects, such as antioxidant, anti-inflammatory, anticarcinogenic, and neuroprotective effects [109–111]. Early in 2005, a study showed that naringenin can significantly protect against cisplatin nephrotoxicity in rats, which is mainly attributed to its antioxidant effect by reducing the depletion of GSH levels and restoring antioxidant enzyme activity, such as SOD, CAT, and GSH-Px, and inhibiting lipid peroxidation [50]. Koyuncu et al. found that naringenin-oxime has a preservative role in the attenuation of cisplatin-induced nephrotoxicity through reducing the oxidative stress, suggesting that the use of naringenin-oxime may enhance the anticancer effect of cisplatin [112].

### 5.5. Lycopene

Lycopene is a natural pigment synthesized exclusively in plants, such as deep-red color of ripe tomato fruits and tomato products [113, 114]. Humans and animals obtain lycopene from dietary sources because they cannot synthesize it [115, 116]. Studies have shown that lycopene has several beneficial effects on human, such as antioxidative, anti-inflammatory, antiatherogenic, and cardioprotective effects [117–120]. Moreover, the level of lycopene in serum tissue is associated with reduced incidence risk of prostate cancer [23, 116, 121]. Here, we discussed whether lycopene could mitigate the renal toxicity induced by cisplatin.

Several studies have shown that lycopene can resist cisplatin-induced nephrotoxicity in rats [72, 122, 123], mainly because lycopene can increase GSH levels, restore the antioxidative enzyme activity, such as catalase, GSH-Px, and SOD, reduce the ROS generation, and alleviate the inflammatory response and lipid peroxidation. Recently, a study showed that lycopene could also suppress cisplatin-induced downregulation of organic anion and cation transporters (OATs and OCTs). It is recognized that OCT1 and OCT2 are responsible for basolateral cation uptake in the proximal tubule, the first step in cation secretion [124], which can inhibit the increase of major efflux transporters, such as multidrug resistance-associated proteins (MRPs), especially inhibiting the MRP2 and MRP4 [82], and improve the ability to protect against the cisplatin-induced nephrotoxicity. Mahmoodnia et al. found that when patients were treated with lycopene tablets (25 mg) and cisplatin administration, the glomerular filtration rate of patients nearly returned to normal baseline [125], indicating that natural antioxidant could be used as clinical strategies in the future.

### 5.6. Resveratrol

Resveratrol (RES) is a phytoalexin present in more than 72 plant species, many of which are consumed by humans, such as grapes, mulberries, and peanuts [126]. Many *in vivo* and *in vitro* studies have shown that resveratrol has a wide range of biological effects, including antioxidant, anti-inflammatory, immune-modulatory, and antitumor effects [127]. In addition, resveratrol has beneficial effects in reducing the risk of cardiovascular diseases [128–130]. Resveratrol has also been shown to have neuroprotective and other chemopreventive activities to scavenge OH, O_2_-, and ONOO- [131, 132]. Several studies demonstrated that resveratrol has the ability to reduce cisplatin-reduced nephrotoxicity. Early in 2008, Amaral et al. reported that resveratrol can play a protective role in reducing cisplatin-induced nephrotoxicity in rats [77] through eliminating the free radicals and inflammatory cell infiltrates. Cigremis et al. also found the same phenomenon and showed that resveratrol can increase the activities of SOD, CAT, and GSH-Px enzyme without changing the mRNA levels of these antioxidant enzymes, suggesting that resveratrol can alter these antioxidative enzyme activities in the posttranslational stage [78]. In 2018, three studies found that resveratrol ameliorates nephrotoxicity caused by cisplatin by promoting anti-inflammation (related to IL-1*β*, TNF-a, and COX- II) and antioxidative stress (i.e., reducing renal tissue MDA and NO levels and increasing GSH concentration and enzymatic activities of SOD and CAT) [133], modulating the cisplatin-pharmacokinetics [83], and activating the ERK signal pathway [134]. However, in 2019, Neag et al. used grape pomace extract (GE) to test protection against kidney damage caused by cisplatin and found conflicting conclusions [135]. Neag et al. found that the GE did not have a protective effect on cisplatin-induced nephrotoxicity; on the contrary, GE enhanced the toxic effect of cisplatin. Although the underlying mechanism remains unknown, it is possible that resveratrol can interact with copper ions, which can cause the synthesis of DNA damage molecules and also increase ROS, and finally counteract the antioxidant effect [136]. This paradox is certainly interesting, suggesting that more researches are needed to clarify the role of resveratrol in the aspect of cisplatin-induced nephrotoxicity.

### 5.7. Fisetin

Fisetin is a bioactive polyphenolic flavonoid, which is widely found in many fruits and vegetables such as persimmons, strawberries, apples, onions, and cucumbers [137]. Several studies reported that fisetin has multiple beneficial pharmacological effects, such as anti-inflammatory and hypolipidemic effect in rheumatoid arthritis [138, 139] and anticancer effect in cervical cancer [71]. A physicochemical analysis research on fisetin revealed that the compound of fisetin as well as its metabolites had a high concentration in mouse kidneys, which indicated its potential in treating renal diseases [140]. When combined with cisplatin treatment, fisetin has also demonstrated a renoprotective effect against cisplatin-induced acute renal injury in rats, and the underlying mechanism was related to the reduced oxidative stress, restored mitochondrial respiratory enzyme activities, and suppressed apoptosis in renal tissue [40]. Moreover, a study suggested that the addition of fisetin to cisplatin treatment could activate both the mitochondrial and cell death receptor pathways and could improve the effect of cisplatin [141] but has not talked about nephrotoxicity. To date, latest researches have shown that fisetin can also reduce cell apoptosis in the kidneys of septic acute kidney injury (AKI) mice induced by lipopolysaccharide by suppressing the NF-*κ*B p65 and MAPK signaling pathways in the kidneys [142]. Ren et al. suggested that fisetin can alleviate renal inflammation through modulating signal transducer and activator of the transcription-3 (STAT3) and transforming growth factor-*β* (TGF-*β*) signaling pathway in hyperuricemic nephropathy mice [143]. In addition, a preclinical study showed that fisetin can be used to build a ROS-activatable nanosystem with active targeting capability for imaging liver and kidney inflammation and for treating liver inflammation [144]. Although these studies did not report the protective effect of fisetin in combination with cisplatin, the exploration of antioxidant and anti-inflammatory mechanisms and even drug delivery methods are very encouraging and could provide certain guidance for the subsequent experimental scheme of combination with cisplatin treatment.

### 5.8. Caffeic Acid Phenethyl Ester (CAPE)

Caffeic acid phenethyl ester (CAPE) is a natural bioactive compound that is extracted from the propolis of honeybee hives [145] and has many protective properties in anti-inflammatory, immunomodulatory, antiproliferative, and antioxidant properties. CAPE has also been shown to inhibit lipoxygenase activities as well as suppress lipid peroxidation [146–150]. In 2004, a study showed that CAPE might reduce the cisplatin-induced renal damage by affecting the antioxidant enzymes, inhibiting NO production, and inhibiting the activation of NF-*κ*B. Two recent articles on neurotoxicity suggested that CAPE can affect the side effects of cisplatin through specific mechanisms, such as influencing nerve growth factor (NGF) through PI3k/Akt and MAPK/ERK pathways and NGF-high-affinity receptors trkA [151, 152], which may provide a new reference for renal injury caused by cisplatin.

### 5.9. Rosmarinic Acid (RA)

RA, a 3,4-dihydroxyphenyllactic acid, is a common natural product with a broad range of applications, such as food preservatives and cosmetics [153]. RA is found to have numerous biological activities, such as antioxidative [154], anti-inflammatory [155], and antitumor effects [156]. The antioxidant activity is mainly due to its phenolic structure, which can easily donate electrons or hydrogen atoms to neutralize free radicals, thus enzymatically recycling the phenoxyl radicals to parent phenolic [157]. Domitrovic et al. found that RA can reduce cisplatin-induced nephrotoxicity by the suppression of oxidative stress, inflammatory response, and apoptotic cell death. Surprisingly, Domitrovic et al. demonstrated that RA has the ability to inhibit cytochrome P450 2E1 (CYP2E1) and HO-1 expression, which can affect the activity of the CYP family of enzymes and increase the production of free radicals, thus resulting in attenuating the oxidative stress induced by cisplatin [158].

### 5.10. Curcumin

Curcumin, a polyphenol insoluble in water, is mostly derived from the rhizomes of Curcuma longa L. [159, 160] and is widely used as a spice and food colorant. Studies have demonstrated that curcumin has antimicrobial, antiviral, anti-inflammatory, antioxidant, and anticarcinogenic effect properties [161–164]. Curcumin structure has two methoxylated phenols and one *β*-diketone group, exhibiting keto-enol tautomerism in solution, which can scavenge ROS, such as H_2_O_2_, singlet oxygen (^1^O_2_), O_2_-, OH, and ONOO− [70]. Several studies showed that pre- and cotreatment (200 mg/kg b. w. p. o. and 15–60 mg/kg b. w. p. o., respectively) of curcumin could alleviate cisplatin-induced renal damage, as evidenced by reduced serum creatinine and BUN levels [165, 166]. The mechanism of these protective effects is related to its ability in scavenging ROS by enhancing antioxidant enzyme activity (CAT, SOD, GPx, and thioredoxin reductase), ameliorating lipid peroxidation and anti-inflammation, and finally rescuing the renal dysfunction induced by cisplatin. Further, curcumin has been reported to enhance cisplatin cytotoxicity in ovarian cancer cells. Although the underlying mechanism may be because polyphenol of curcumin can increase cisplatin cellular concentration and binding to DNA [167], more *in vivo* experiments are needed to explore whether curcumin can really enhance the efficacy and reduce toxicity caused by cisplatin.

### 5.11. Origanum majorana

*Origanum majorana* L. is a member of the mint family Lamiaceae, which is mainly distinguished by its common names, such as oregano and sweet marjoram [168]. Origanum plants can exhibit a wide range of biological activities, such as anticancer, antidiabetic, antinociceptive, antimicrobial, insecticidal, hepatoprotective, cytotoxicity, and antilipase properties [169], can have great ferric reducing activity, and can display enhanced antioxidant activity [170]. It is regarded as generally safe by the Food and Drug Administration [171]. Two previous studies reported the protective effect of the Origanum majorana ethanolic extract (OMEE) through reducing the cisplatin-induced nephrotoxicity: Sayed et al. verified the antioxidant activity of OMEE through inhibiting DPPH (2, 2-diphenyl-1-picrylhydrazyl) free radical [168], and Soliman et al. showed that OMEE can alleviate kidney damage from significant decreasing in MDA and NO levels, increasing in each of GSH, and restoring the SOD and CAT activity as well [27]. In addition to cisplatin, the oxidative stress and inflammation induced by dose-dependent gentamicin can be alleviated by marjoram extract [172]. Another Origanum species, *Origanum vulgare* leaf extract, also showed the nephron-protective ability on gentamicin-induced nephrotoxicity in a rat model, through increasing the levels of renal SOD, CAT, and vitamin C and decreasing the expression of renal MDA and TNF-*α* gene [173]. Therefore, the above studies indicated that Origanum species are promising compounds to resist oxidative stress induced by cisplatin.

### 5.12. Others

After reviewing a large number of literatures on the protective ability of natural antioxidants against renal toxicity of cisplatin, we found that, besides the natural antioxidants mentioned above, some sporadic natural antioxidants, such as betaine, hyperin, formononetin, and Camellia sinensis leaf buds, also showed the property on protecting the kidney from damage caused by cisplatin, but related studies are few in number. Betaine, a natural component, also called trimethyl glycine, is widely found in plants, microorganisms, and other rich dietary sources [174]. One study showed that betaine also have the ability to protect against cisplatin-induced oxidative stress due to its free radical scavenging effect and it can increase the content of GSH, as well as suppress inflammation, NF-*κ*B activation, and apoptosis during cisplatin toxicity [52]. Hyperin, a flavonol extracted from the Chinese herb Abelmoschus manihot L. Medic, is mainly found in the south of China. A study showed that the possible mechanism of hyperin attenuating cisplatin-induced kidney injury was associated with the inhibition of inflammatory and oxidant responses by inhibiting NF-*κ*B and activating Nrf2 signaling pathways [175]. Formononetin, an O-methylated isoflavone, is one of the major compounds in red clover plants. A study demonstrated that formononetin is an efficient protectant against cisplatin-induced cell death through inhibiting intracellular ROS accumulation in pig kidney epithelial LLC-PK1 cells, and its mechanism is related to the properties in alleviating ROS accumulation and antiapoptosis, without altering the antitumor ability of cisplatin [176]. Camellia sinensis leaf buds (CSB) and its flowers (CSF) can restore renal dysfunction, reduce the degree of lipid peroxidation, and suppress antioxidant range to alleviate cisplatin-induced renal toxicity [177].

In brief, many other natural antioxidants that are capable of resisting cisplatin nephrotoxicity and the protective effect can be achieved by multiple mechanisms at multiple levels and thereby deserved further exploration.

## 6. The Other Toxicities Induced by Cisplatin and Its Natural Antioxidant Protection

It is commonly known that, beyond the nephrotoxicity, cisplatin can also frequently induce other side effects, such as ototoxicity and hepatotoxicity. Ototoxicity is one of the most frequent side effects in cisplatin, with the incidence of 11%-97%. It usually begins with a symmetrical and irreversible sensorineural hearing loss, related to the dose of cisplatin used, and has a property of accumulation [178]. The exact mechanism of cisplatin-induced ototoxicity is unclear. It was reported that the transporters (e.g., OCT2), the transient receptor potential channel family members, and other calcium channels, chloride channels, as well as the excessive accumulation of ROS can lead to ototoxicity [179, 180].

Another common side effect is hepatotoxicity, which is usually observed following the administration of large cisplatin doses [181] or small repeated doses [182, 183]. The mechanism of hepatotoxicity is not accurately understood as well. Previous studies suggested that hepatotoxicity might be related to oxidative damage to cardiolipin and proteins with sulfhydryl groups and hepatic cell death by apoptosis via mitochondria as nephrotoxicity and the altered energy metabolism in the liver [184]. However, several studies demonstrated that the main dose-limiting side effect is renal toxicity [15–18], transporters related to cellular uptake of cisplatin are highly expressed in the proximal and distal tubules of the kidneys [185], and the rate of elimination of cisplatin is about 25% within just 24 h and 50% within 5 days in which more than 90% of total excretion occurs through renal excretion [186]; thus, renal excretion is the principal route of excretion of cisplatin, leading to major accumulation in the kidneys and consequent nephrotoxicity. Toxicity induced by cisplatin, excessive ROS accumulation, and the reaction in multiple organ cells have a similar mechanism. Summarizing previous researches, we observed that, besides suppressing the nephrotoxicity, several natural antioxidants also have the ability to protect against other side effects. For example, CAPE has the hepatoprotective properties on cisplatin-induced damage [181, 187], and rosmarinic acid can ameliorate the liver and testicular toxicity [188]. Our review suggests that natural products may exhibit wide use in decreasing multiple toxicity or side effects induced by cisplatin.

## 7. The Implications and Challenge in Clinical Trials

After discussing the advantages of natural antioxidants, some implicated strategies need to be considered in clinical application. It is well recognized that the three platinum drugs commonly used in the current treatment of cancer are cisplatin, carboplatin, and oxaliplatin. Cisplatin, the first platinum anticancer drug, and carboplatin are the most common second-generation drugs; oxaliplatin is the third one. Cisplatin became high profile not only for its therapeutic response but also because of its severe side events. The second-generation platinum has a lower aquation rate due to the bidentate cyclobutane dicarboxylate ligand [189]. Because of reduced reactivity, the neurotoxicity and ototoxicity after carboplatin treatment are much less pronounced. As a result of reduced toxicity profile, carboplatin is suitable for more aggressive high-dose chemotherapy [190]. The third-generation platinum was developed to overcome resistance against cisplatin and carboplatin. Organic cation transporters, such as OCT1 and OCT2, have been revealed to mediate oxaliplatin uptake, as their overexpression significantly increases the cellular accumulation of oxaliplatin, but not cisplatin. Colorectal cancer cells overexpress organic cation transporters, resulting in the high therapeutic effect of oxaliplatin in this specific cancer type [191]. Therefore, the second generation of platinum drugs can be used in the treatment combined with natural antioxidant regardless of dosage, which might directly reduce the adverse events induced by platinum and even cure the disease. Additionally, it should be noted that the OCT2 expression can be regulated by the treatment of oxaliplatin, but also some antioxidants. Therefore, the selection of drug is essential. In this situation, it is necessary to clarify whether the therapeutic and side effects will be enhanced or weakened when platinum is combined with antioxidants.

DNA was identified as the major cellular target of platinum. Cisplatin impairs normal DNA functions by generating monoadducts as well as DNA crosslinks, forming DNA adducts, and activating various signal-transduction pathways, such as DNA-damage recognition and repair, cell cycle arrest, and programmed cell death/apoptosis [192]. Different DNA damage responses (DDR) in normal and cancer cells provide a useful explanation for the initial antitumor effect of platinum drug. Among the DDR mechanisms, the homologous recombination (HR) pathway is of particular interest because it mediates the error-free repair of double-strand breaks, which are highly toxic to cells. In some cancer cells, BRCA1 mutations and other HR defects are enriched and platinum drugs show a significant benefit [193]. In this situation, these different characteristics between normal kidney cells and malignant cancer cells can be a protective strategy applied in cisplatin treatment, because the antitumor mechanism in HR-deficient cancer cell can be largely dependent on the irreparable DNA damage and error repair, rather than ROS accumulation. It provides an ideal approach to protect the normal kidney cell, enhance the therapeutic effects of cisplatin in cancer cells, and provide subtypes of disease model in the application of natural antioxidant in protecting against cisplatin-induced nephrotoxicity in the future.

As mentioned above, several preclinical models demonstrated that natural antioxidants have the properties in attenuating cisplatin-induced nephrotoxicity. These studies may not be considered as strong evidence for adjuvant clinical strategies in cisplatin treatment. Although the preclinical models reported the use of cardamonin, quercetin, and resveratrol combined with high-dose cisplatin in animals, most cancer patients in clinical practice are treated with low cisplatin doses, and the interference of most of these natural agents in the antitumor activity of cisplatin remains unknown. Therefore, whether these experiments can be extrapolated in the clinical practice remains unclear. However, these studies provide better understanding of the underlying mechanism that natural antioxidant can attenuate cisplatin-induced nephrotoxicity. A study even demonstrated that lycopene can attenuate cisplatin treatment by inducing kidney damage in human [125]. Cardamonin, quercetin, naringenin, fisetin, and curcumin (Table 1) also displayed the ability to improve the anticancer effect of cisplatin when cisplatin was combined with these natural supplements. Actually, ROS also is a common factor of cisplatin therapeutic effects, of which excessive accumulation can influence both the viabilities of the normal cell and cancer cell, but these preliminary experimental evidences suggest that natural antioxidants can protect the kidney cells yet enhance the antitumor effect of cisplatin in cancer cells, although the exact mechanism is still unclear. Therefore, these studies revealed the effect of natural components combined with cisplatin *in vivo* and *in vitro* and discussed the possibility preliminary on adding these natural antioxidants as an adjuvant therapy in cisplatin treatment, but more preclinical trials with high quality are still needed to validate the usefulness of these agents in attenuating the cisplatin-induced kidney damage.

## 8. Summary

The clinical application of cisplatin has been limited by its toxic and side effects; thus, eliminating or ameliorating these toxicities is crucial for treatment success. The most common side effect is renal toxicity. Researches showed that the mechanism of kidney damage caused by cisplatin is related to the promotion of apoptosis and necrosis of renal cells and tubules, such as vascular dysfunction, immune response, and other reactions. Among the mechanism of kidney injury, accumulation of ROS and oxidative stress might be the upstream reactions. ROS are the early signaling molecules that can subsequently provoke apoptosis effect and inflammatory response and cause different degrees of damage to the intracellular material components. The mechanism of oxidative stress induced by cisplatin is mainly attributed to the consumption of endogenous antioxidants, destruction of antioxidant enzymes, induction of mitochondrial damage, and destruction of the CYP system, which result in the excessive accumulation of ROS in cells (Figure 1). Experiments have shown that natural antioxidants have a certain protective effect against cisplatin nephrotoxicity damage (Table 1). Natural antioxidants can eliminate free radicals, maintain the stability of endogenous antioxidants, restore antioxidant enzyme capacity, and protect against mitochondrial damage to achieve resistance to ROS induced by cisplatin, improve subsequent nonredox pathways, improve the filtration rate of cisplatin in the kidney, and modulate cisplatin pharmacokinetics and can eventually protect the kidney from toxicity (Figure 2). Meanwhile, studies have shown that the combined use of natural antioxidants with cisplatin can also increase the anticancer effect of cisplatin [75, 104, 141]. In summary, a large number of experimental studies of natural antioxidants on cisplatin nephrotoxicity provided abundant preclinical data for the clinical use of cisplatin and proposed a new idea for the detailed mechanism of cisplatin nephrotoxicity. In order to comprehensively explore the adverse events of cisplatin and the efficacy of these natural compounds in clinical practice, more preclinical and clinical studies are certainly urgently needed.

## Figures and Tables

**Table 1:** Major natural products reducing cisplatin-induced nephrotoxicity in different experimental models.

Antioxidant	Experimental evidence		Effects on cancer therapy
	*In vivo*	*In vitro*	
Hesperidin	Mice [92], rat [53, 91, 194]	HK2 (kidney, *Homo sapiens*) [92]	Unclear
Cardamonin	Rat [75]	—	Improved [75]
Quercetin	Rat [76, 102–105]	LLC-PK1 (kidney, *Sus scrofa*) [101]	Improved [104]
Naringenin	Rat [50, 112]	—	Improved [112]
Lycopene	Rat [72, 82, 122, 123], human [125]	—	Unclear
Resveratrol	Mice [133], rat [77, 83, 134], rabbit [78]	—	Unclear
Fisetin	Rat [40]	—	Improved [141]
Caffeic acid phenethyl ester (CAPE)	Rat [73]	—	Unclear
Rosmarinic acid (RA)	Rat [158]	—	Unclear
Curcumin	Rat [165, 166]	—	Improved [167]
Origanum majorana	Rat [27]	—	Unclear
Betaine	Rat [52]	—	Unclear
Hyperin	Rat [175]	—	Unclear
Formononetin	—	LLC-PK1 (kidney, *Sus scrofa*) [176]	Unclear
